# Diuretics: a contemporary pharmacological classification?

**DOI:** 10.1007/s00210-022-02228-0

**Published:** 2022-03-16

**Authors:** Miriam C. A. Kehrenberg, Hagen S. Bachmann

**Affiliations:** Institute of Pharmacology and Toxicology, Centre for Biomedical Education and Research, Witten/Herdecke University, Witten, Germany

**Keywords:** Diuretics, Nomenclature, Carbonic anhydrase inhibitors, Acetazolamide, Pharmacological classification

## Abstract

Diuretics are drugs that increase the flow of urine. They are commonly used to treat edema, hypertension, and heart failure. Typically, the pharmacological group consists of five classes: thiazide diuretics, loop diuretics, potassium-sparing diuretics, osmotic diuretics, and carbonic anhydrase inhibitors. This traditional classification and the nomenclature of diuretics have not changed over the last decades, which means that it was not adapted to current pharmacological research. Modern approaches in the field of pharmacological nomenclature suggest the introduction of mechanism-based drug class designations, which is not yet reflected in the group of diuretics. Moreover, included drug classes have lost their relevance as diuretic agents. Carbonic anhydrase inhibitors, for example, are mainly used in the treatment of glaucoma. Newer agents such as vasopressin-2 receptor antagonists or SGLT2 inhibitors possess diuretic properties but are not included in the pharmacological group. This review discusses the currentness of the pharmacological classification of diuretics. We elaborate changes in the field of nomenclature, the contemporary medical use of classical diuretics, and new diuretic agents.

**Supplementary Information:**

The online version contains supplementary material available at 10.1007/s00210-022-02228-0.

## Introduction

Diuretics are among the most frequently prescribed medications (Ellison 2019). They are defined as drugs promoting the excretion of water and electrolytes by the kidneys (Aktories et al. 2017; Buckingham 2020) and thereby increasing the rate of urine flow. Buildup of fluid in body tissues is a result of an inability of the kidneys to release sodium and the water that is excreted along with it. According to standard textbooks in the field of pharmacology, diuretics are used for the treatment of edema, heart failure, or hypertension (Aktories et al. 2017; Buckingham 2020). There are many compounds exhibiting diuretic effects in the human body, but traditionally, five classes of drugs are categorized and taught as diuretics. These five classes are carbonic anhydrase inhibitors (CAIs), loop diuretics, osmotic diuretics, potassium-sparing diuretics, and thiazides (Aktories et al. 2017; Buckingham 2020; Lüllmann et al. 2016; Vardanyan and Hruby 2006; Wile 2012). Table 1 gives an overview of these compounds.

**Table 1:** Traditional classes of diuretics

Drug class	Mechanism	Molecular site of action	Target site nephron	Lead compound (s)	Potassium excretion	Main medical indications of lead compound
Carbonic anhydrase inhibitors	Natriuresis	Carbonic anhydrase	Proximal tubule	Acetazolamide	K^+^ ↑	Glaucoma; epilepsy, high-altitude disorders
Loop diuretics	Natriuresis	Na^+^-K^+^-2Cl^−^ symporter	Loop of Henle	Furosemide	K^+^ ↑	Edema associated with heart failure, oliguria, hypertension
Osmotic diuretics	Osmosis	Unspecific	Everywhere	Mannitol	K^+^ ↑	Acute renal failure, cerebral edema, glaucoma
Potassium-sparing diuretics	Natriuresis	1. Epithelial Na^+^ channels 2. Mineralocorticoid receptor	Late distal tubule + collecting duct	Triamterene Spironolactone	K^+^ ↓	Triamterene: add-on diuretic to reduce risk of hypokalemia, hypertension Spironolactone: heart failure, refractory edema, add-on diuretic to reduce risk of hypokalemia
Thiazides	Natriuresis	Na^+^-Cl^−^ symporter	Distal convoluted tubule	(Hydrochloro)-thiazide	K^+^ ↑	Hypertension, edema, heart failure, hypercalciuria

↑increased; ↓decreased. Classification and major indications are according to Martindale: The Complete Drug Reference (Buckingham 2020)

According to Table 1, it becomes apparent that the nomenclature is not consistent within this pharmacological group. Depending on the type of drug, nomenclature is based on mechanism (osmotic diuretics), molecular site of action (CAIs), target site of nephron (loop diuretics), lead compound (thiazides), or potassium excretion (potassium-sparing diuretics).

Furthermore, varying indications within this pharmacological group are striking. Interestingly, therapeutic areas do not always match the indications listed in pharmacology textbooks for diuretics (heart failure, edema, or hypertension). This is illustrated, for example, by the indications of mannitol as osmotic diuretic (e.g., high intracranial pressure or glaucoma), but this is especially true for CAIs. Acetazolamide as lead compound of CAIs is mainly used in the treatment of ophthalmological diseases like glaucoma. Epilepsy or high-altitude disorders are further medical indications. Apart from that, newer drugs are not considered in the classification of diuretics as defined in pharmacology textbooks, but diuretic effects are also known, e.g., for SGLT2 inhibitors (Delanaye and Scheen 2021; Titko et al. 2020).

New research findings in drug discovery, newly identified mechanisms of action, and drug repurposing can render historical pharmacological classifications obsolete. This review discusses the validity of the pharmacological classification of diuretics including its nomenclature, its currentness, and its completeness. Particular focus is on CAIs and its lead compound acetazolamide.

## Main analysis

### Nomenclature of diuretics

Molecular targets of drug classes were often not known at the time the drugs were launched. That is why, in many cases, traditional drug designations are not mechanism based but possess a descriptive character (Seifert 2018; Uchida 2018). The fact that these traditional terms are often scientifically imprecise has already been discussed in recent articles (Caraci et al. 2017; Seifert 2018; Seifert and Schirmer 2020, 2021a, b; Zohar et al. 2014). The term potassium-sparing diuretic, for example, does not reflect the molecular target of this drug class that can be either the epithelial Na^+^ channel or the mineralocorticoid receptor. Instead, the term describes a characteristic that distinguishes these drugs from other diuretic agents. The terms loop diuretics and thiazides are also derived from specific properties (target site of nephron and name of lead compound) but do not reflect the drug target. As a result of this impreciseness, the drug chlorthalidone, for example, is often called “thiazide like.” This classification of chlorthalidone is misleading, because not only the chemical structure is different but also some of its molecular and clinical effects differ significantly from thiazides (Kurtz 2010). Osmotic diuretics do not act via specific receptors or channels but mechanistically via osmosis. Therefore, a designation that includes the attribute “osmotic” is scientifically correct. Only the term carbonic anhydrase inhibitor properly reflects the mechanism of action, namely the enzyme that is inhibited.

### Medical indications of traditional diuretics

In Table 1, the main medical indications of the lead substances of each diuretic class are illustrated. The indications listed for loop diuretics, potassium-sparing diuretics, and thiazides are typically associated with diuretics; those from osmotic diuretics and CAIs at least partly deviate.

Mannitol is a sugar alcohol. After parenteral administration, it raises the osmotic pressure of the blood plasma. Consequently, water is drawn out of the tissues leading to diuresis (Buckingham 2020). Mannitol does not possess a specific biological target such as a receptor or enzyme but the ability to induce the movement of extravasal water to the vessels. All of its medical uses can be ascribed to its osmotic effect. The drug can be administered for osmotic diuresis, to reduce intracranial pressure, cerebral edema, or intraocular pressure (glaucoma).

In contrast to mannitol, CAIs have a defined molecular target. They act as enzyme inhibitors of the carbonic anhydrase (CA). CAs are found in various tissues, including but not limited to the renal tissue (Lindskog 1997). As shown in Table 1, this is reflected in the medical use of CAIs (here: acetazolamide) and raises questions about the pharmacological classification of this drug class.

### Classification of CAIs and acetazolamide

Acetazolamide is the prototype of CAIs, already launched as diuretic in 1954 (Supuran 2021). Carbonic anhydrases are abundant in the proximal tubule. Acetazolamide administration results in alkaline diuresis, but its diuretic effect is only weak. Its long-term usefulness is limited by the development of metabolic acidosis (Aslam and Gupta 2021). CAIs also suppress aqueous humor formation in the eyes leading to a reduction of intraocular pressure. This explains the clinical use of CAIs in the treatment of glaucoma being associated with an elevated intraocular pressure (Aktories et al. 2017; Brunton et al. 2017; Buckingham 2020; Masini et al. 2019).

Interestingly, the classification of CAIs differs depending on pharmacology textbook. Historically, CAIs were categorized as diuretics, but some textbooks meanwhile classify them as antiglaucoma drugs. In the textbook Martindale: The Complete Drug Reference (Buckingham 2020), an international pharmacology standard reference, CAIs are mentioned in the diuretics section but introduced and discussed in the section of antiglaucoma drugs. Other standard references like Goodman and Gilman’s Pharmacological Basis of Therapeutics (Brunton et al. 2017) and Basic & Clinical Pharmacology (Katzung and Vanderah 2020) present the classical division; CAIs are described in the section of diuretics. The same classification (as diuretics) can be found in standard pharmacology textbooks in Germany (Aktories et al. 2017). The World Health Organization published an online brochure “Learning clinical pharmacology using INN stems” (WHO 2018) with the aim to classify medicines in a systematic way. The brochure is intended for healthcare professionals. It lists CAIs in the section of diuretics, but a note or more specifically a warning is added that this drug class is mainly used to reduce intraocular pressure in glaucoma (WHO 2018).

To further investigate the appropriateness of the pharmacological classification of CAIs as diuretics or as antiglaucoma drugs, we checked the approved indications of acetazolamide preparations (as lead compound of CAIs). Table 2 shows the approved indications of acetazolamide single-ingredient preparations in different countries. In each listed country, there exists an acetazolamide preparation for the treatment of glaucoma. Furthermore, in some countries (e.g., Czech Republic, Denmark, or Germany), this is the only approved indication. Other indications (e.g., epilepsy, high-altitude disorders, and edema) vary depending on preparation and country. It is noticeable that the medical purposes that would pharmacologically be assigned to diuretics are diuresis (approved indication in only one of ten countries listed) and edema (in five of ten countries listed).

**Table 2:** Indications of acetazolamide single-ingredient preparations in different countries

Country	Preparations	Indications
Brazil	Diamox®	Epilepsy; glaucoma; altitude sickness
Czech Republic	Diluran®	Glaucoma
Denmark	Diamox®	Glaucoma
Germany	Acemit®, Glaupax®	Glaucoma
India	Acetamide®, Acetamin®, Acetariv®, Avva®, Diamox®, Iopar®	Diuresis, glaucoma, epilepsy, high-altitude disorders
Israel	Uramox®	Glaucoma; edema; acute mountain sickness
Japan	Diamox®	Glaucoma; epilepsy; respiratory acidosis; edema; Ménière’s disease
New Zealand	Diamox®, Glaumox®	Epilepsy; glaucoma; edema
Switzerland	Diamox®, Glaupax®	Glaucoma; edema; respiratory insufficiency; epilepsy; pancreatic disorders; altitude sickness
United Kingdom	Diamox®, Eytazox®	Glaucoma, edema, epilepsy

Preparations are according to online version of Martindale: The Complete Drug Reference (Royal Pharmaceutical Society 2021)

### PubMed analysis of CAIs and acetazolamide publications

**Fig. 1:**
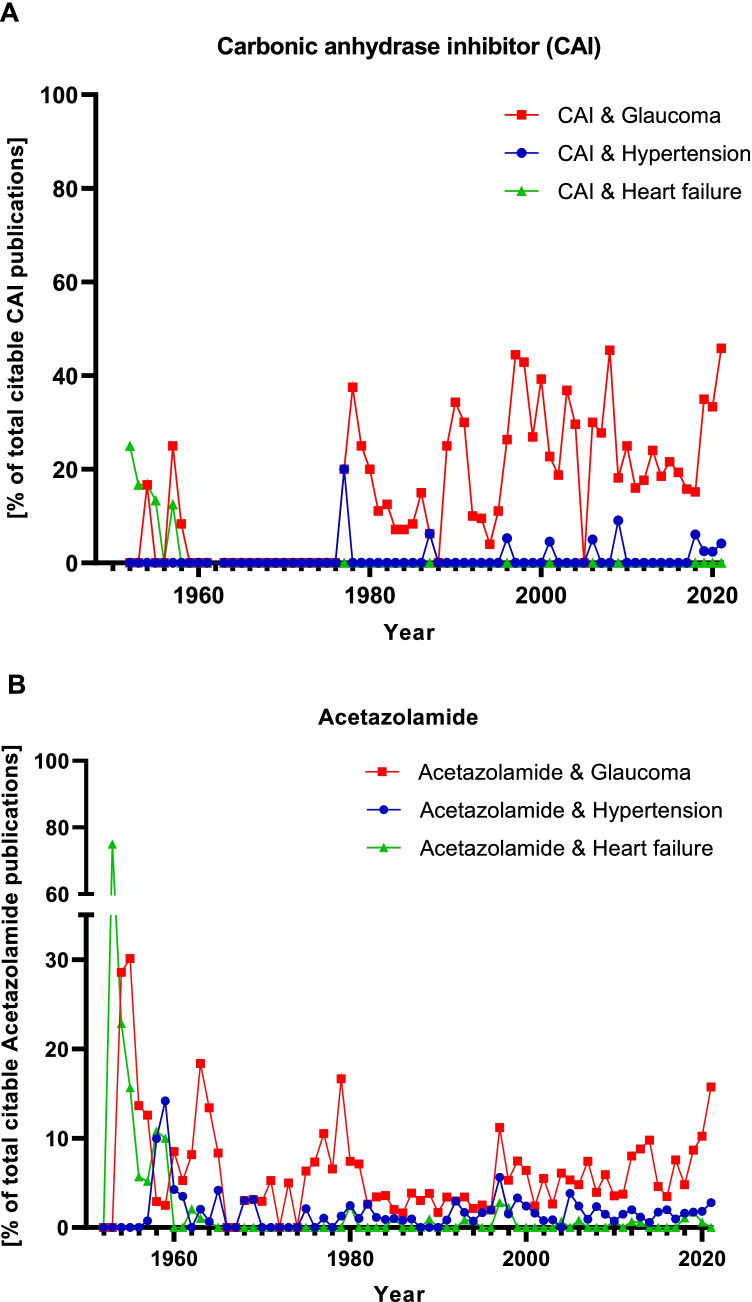
Citation frequency of the medical indications of hypertension, heart failure, and glaucoma in the context of carbonic anhydrase inhibitors. PubMed searches were limited to titles and abstracts of articles. The additional filter “Journal Article” was applied, and the journal category was set to “MEDLINE.” The ratio per year is shown in percent. The ratio is defined as number of articles mentioning A: all CAIs and B: acetazolamide together with the medical indications of hypertension, heart failure, or glaucoma relative to the total number of articles citing CAIs and acetazolamide, respectively

In order to get to know which of the two therapeutic uses (diuretic drug or antiglaucoma drug) is prominent in publications mentioning CAIs or acetazolamide, we analyzed scientific articles indexed in PubMed. We selected two medical indications typically associated with diuretics (hypertension and heart failure) and glaucoma as specific indication for CAIs (Presne et al. 2007; Reyes 2002). The ratio per year of each medical indication is shown in the graphs in Fig. 1A for CAIs and Fig. 1B for acetazolamide. Of the three indications analyzed, glaucoma is most frequently mentioned together with CAIs and acetazolamide, respectively. Typical indications of diuretics (hypertension, heart failure) are rarely mentioned.

### Clinical trials with acetazolamide drug intervention

**Fig. 2:**
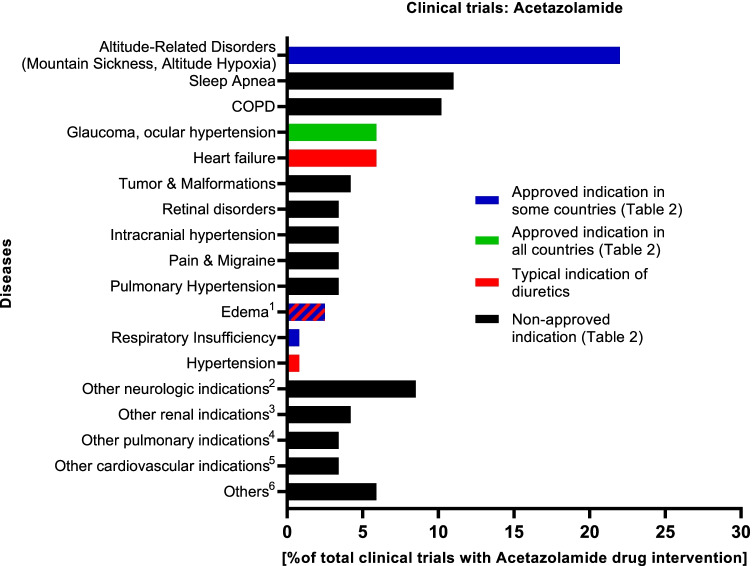
Clinical trials with acetazolamide drug intervention. Relative share by conditions. The search on clinicaltrials.gov was performed on October 14, 2021. In total, 118 clinical trials with acetazolamide drug intervention were found for various conditions. The proportion relative to the total number of clinical trials with acetazolamide drug intervention is shown in percent. ^1^Edema: cerebral/macular/pulmonal edema, nephrotic syndrome. ^2^Other neurologic indications: acute cerebrovascular accident, aneurysma, cerebral vasospasm, cerebrospinal fluid leak, cognitive investigation, Landau-Kleffner syndrome, multiple sclerosis, neurocysticercosis, normal pressure hydrocephalus, schizophrenia, status epilepticus, subarachnoid hemorrhage. ^3^Other renal indications: Bartter syndrome, cisplatin nephrotoxicity, contrast-induced nephropathy, kidney calculi, obesity-induced hyperfiltration. ^4^Other pulmonary indications: alkalosis, hypercapnia, hypoxia, respiratory insufficiency, ventilator weaning. ^5^Other cardiovascular indications: Andersen-Tawil syndrome, chronic orthostatic intolerance, orthostatic hypotension, tachycardia, thoracic aneurysm. ^6^Others: cocaine use, *H. pylori* infection, PMM2-CDG (Jaeken syndrome), sickle cell disease, Thalassemia, validation of method to obtain the arterial input function

In order to go further into detail, we assessed clinical trials with acetazolamide drug intervention. The results are illustrated in Fig. 2. Looking at the indications we focused on so far (hypertension, heart failure, and glaucoma), neither these typical diuretic indications nor glaucoma has the largest share. The percentage for “heart failure” is approximately 6% and is comparable to the ratio of “glaucoma.” The share of clinical trials for hypertension is very low, namely less than 1%. Largest shares have other indications like altitude-related disorders, sleep apnea, and chronic obstructive pulmonary disease (COPD). As shown in Table 1 and in Table 2, high-altitude disorders as indication are both one of the main medical indications of acetazolamide and one of the approved indications for single-ingredient preparations. Sleep apnea or COPD do not meet these criteria. Across the 118 trials found, there are many other indications beside sleep apnea and COPD that are outside the scope of the original medical purposes of diuretic agents. Pain and tumors are only two further examples. Analysis of clinical trials reveals acetazolamide as candidate for drug repurposing which renders the pharmacological classification of acetazolamide and CAIs even more complex. In case of drug repurposing, new therapeutic uses are identified for drugs that were already approved for other indications (Oprea et al. 2011, Oprea and Mestres 2012; Pushpakom et al. 2019). As safety data, pharmacokinetic and manufacturing data are already available; this approach can accelerate the process of finding new therapies for, e.g., rare diseases or cancer with lower clinical risks and lower costs (Parvathaneni et al. 2019; Sleire et al. 2017; Talevi 2021).

### Therapeutic areas of CAIs and acetazolamide

Looking at the prescription volumes of acetazolamide in Germany, we see that the volumes increased in 2020 and 2019 by more than 10% compared to the previous year (Ludwig et al. 2021). Acetazolamide preparations on the German market are for systemic application (tablets) approved for the treatment of glaucoma (see Table 2). In long-term therapy, local application of CAI preparations (e.g., dorzolamide) is predominant. Therefore, the increasing prescription volumes of acetazolamide preparations indicate that the use of this drug goes well beyond the traditionally assigned indications.

Indeed, research in the field of pharmacology proves that the clinical benefit of CAIs is not limited to their diuretic action or to their use in the treatment of glaucoma (Van Berkel and Elefritz 2018). Some indications for which CAIs are studied were already mentioned in this article; some further therapeutic areas will be described in the following paragraph.

Acetazolamide is known to rapidly reduce pulmonary and cerebral edema. That is why it is approved for the treatment of high-altitude disorders in, e.g., Brazil, India, Israel, or Switzerland (Table 2). Moreover, it is used for the prophylaxis of acute mountain sickness (Buckingham 2020; Low et al. 2012).

The administration of acetazolamide in the management of epilepsy already dates back to the 1950s (Hoddevik 2000). Either alone or with other antiepileptic drugs, it may be given to treat different forms of epilepsy, e.g., refractory partial seizures with or without secondary generalization (Ozsoy 2021). Its effect is believed to be due to inhibition of CAs in glial cells. However, chronic use is associated with a rapid development of tolerance (Buckingham 2020).

Due to its property to inhibit CA isoforms involved in cerebrospinal fluid secretion, acetazolamide is an effective agent in the treatment of idiopathic intracranial hypertension (Supuran 2015; Wall 2017). Acetazolamide use leads to a decreased cerebrospinal fluid secretion.

COPD is one of the conditions most frequently studied in clinical trials with acetazolamide intervention (Fig. 2). CAIs are relevant for patients affected by respiratory failure with a concurrent metabolic alkalosis. The drugs may have favorable effects due to their ability to inhibit the reabsorption of bicarbonate ions from renal tubules leading to a decrease in pH (Cole et al. 2019; Heming et al. 2012; Tanios et al. 2018).

Another potential therapeutic area not yet mentioned here is cancer. There are ongoing clinical trials testing the effectiveness of acetazolamide in the treatment of oncological diseases (clinicaltrials.gov, accessed October 14, 2021); for example, a phase I trial for patients suffering from localized small cell lung cancer (ClinicalTrials.gov Identifier: NCT03467360). Another trial investigates the effectiveness of acetazolamide together with the cytostatic drug temozolomide in adults with malignant glioma (ClinicalTrials.gov Identifier: NCT03011671).

Moreover, neuroprotective properties of CAIs are investigated that would render them interesting drugs for the treatment of Alzheimer’s disease. In animal models, it has been shown that formation of amyloid beta can be potentially prevented due to their administration (Supuran 2021).

There are various other indications not usually associated with CA inhibition that are discussed in literature for the use of CAIs. These include, e.g., obesity, neuropathic pain, cerebral ischemia, oxidative stress, rheumatoid arthritis, and infectious diseases (Supuran 2021). The fact that CAIs are studied for such a wide variety of diseases underlines the need to rethink their pharmacological classification. CAs play a key role in numerous physiological processes which we already demonstrated by presenting several potential therapeutic areas of CAIs. But, of course, this can also be illustrated by analyzing characteristic side effects. Patients undergoing an acetazolamide therapy report, e.g., taste disturbances including abnormal taste of carbonated drinks “champagne blues” (Graber and Kelleher 1988). Due to this characteristic side effect of CAIs, CA involvement in the sense of taste was assumed. A specific CA isoform was identified to be required for taste response to carbon dioxide (CA IV); another isoform (CA VI) is reported to be a salivary enzyme playing an essential role in the sense of taste and smell (Chandrashekar et al. 2009; Dunkel and Hofmann 2010; Patrikainen et al. 2014).

### Further drug classes with diuretic action

Our analysis up to now demonstrates that the five traditional diuretic drug classes are not homogeneous both in nomenclature and in their medical use. Another raised question concerns the completeness of this pharmacological group.

Many more substances are known to be diuretically active ranging from dietary components to herbal ingredients to recently launched drugs. The two most popular diuretic substances are commonly found in diet: alcohol and caffeine. Mechanistically, alcohol inhibits the release of vasopressin, an antidiuretic hormone, leading to increased fluid loss (Hobson and Maughan 2010; Polhuis et al. 2017). Vasopressin plays a key role in the regulation of water excretion. Its effect on the collecting ducts of the kidneys leads to reabsorption of solute-free water to the plasma resulting in a reduced urine volume. Normally, rising plasma osmolality stimulates vasopressin secretion. Caffeine also induces a mild diuretic effect (Maughan and Griffin 2003). Caffeine, as well as theophylline and theobromine, chemically belong to methylxanthines. The underlying mechanisms of their diuretic action are not yet fully understood. It is suggested to be related to their properties as adenosine receptor antagonists and phosphodiesterase inhibitors in the proximal tubule of the kidneys (Osswald and Schnermann 2011; Zhang et al. 2015).

Herbal remedies have a long tradition in the history of mankind. In the field of phytotherapy, a vast number of medicinal plants are known to possess diuretic or aquaretic effects, such as *Betula pendula* (birch) leaves (Raal et al. 2015), *Urtica dioica* (stinging nettle) herb, *Solidago* (goldenrod) herb (Yarnell 2002), or *Orthosiphon stamineus* leaves (Adam et al. 2009). Their diuretic properties originate from secondary plant metabolites including flavonoids (Vargas et al. 2018) and saponins (Rhiouani et al. 1999).

Newer drugs have also not been adequately included in the classification of diuretics as currently presented in textbooks. Tolvaptan is one of the newer diuretic drugs that was approved by the FDA back in 2009 (https://www.accessdata.fda.gov/, accessed 7 December 2021). It is an oral vasopressin-2 receptor antagonist causing free water excretion (aquaresis). As tolvaptan corrects low serum sodium levels, the drug is used to treat euvolemic and hypervolemic hyponatremia (Buckingham 2020; Plosker 2010).

Another example is sacubitril, a neprilysin inhibitor. The combination of sacubitril/valsartan (AT1R antagonist) was approved by FDA in 2015 (https://www.accessdata.fda.gov/, accessed 8 December 2021). Sacubitril is a prodrug being metabolized to sacubitrilat that inhibits the neutral endopeptidase neprilysin. Through neprilysin inhibition, the breakdown of several endogenous vasoactive peptides is decreased such as natriuretic peptides, bradykinin, substance P, adrenomedullin, and vasoconstrictor peptides. Sacubitril/valsartan administration results in vasodilatation and reduction in blood volume but also in increased sodium and water excretion via the kidneys (D'Elia et al. 2017). It is used for the treatment of heart failure (Buckingham 2020).

Sodium glucose cotransporter 2 (SGLT2) inhibitors are also a relatively new class of drugs to be considered in this context. They are used in the management of diabetes mellitus as SGLT2 inhibition induces glycosuria by suppressing renal reabsorption of glucose. But, these drugs also inhibit proximal sodium absorption and therefore possess diuretic properties. That is why these drugs have already been included in some of the current reviews of diuretics (Mullens et al. 2019).

## Conclusion

The classification of diuretics that is typically integrated in pharmacology textbooks has historically evolved and includes five drug classes: CAIs, loop diuretics, osmotic diuretics, potassium-sparing diuretics, and thiazides. The nomenclature within this group has a descriptive character and does not follow consistent rules. Although typical indications of diuretics are clearly defined in pharmacology textbooks, the therapeutic areas of these five drug classes do not consistently match these indications. This mismatch is particularly noticeable for CAIs; it was therefore examined in more detail. In fact, the classification of CAIs as diuretics is most common, but they may also be categorized as antiglaucoma drugs. Glaucoma is the only indication that is approved for the lead compound of CAIs in all countries we studied, and glaucoma is more often cited in articles mentioning CAIs compared to diuretic indications. But, an appropriate categorization of CAIs is more complex due to many other therapeutic areas that are clinically relevant.

Beside the example of CAIs, the entire group of diuretics harbors inconsistencies not only with regard to nomenclature and medical use but also in relation to its completeness. A lot of other diuretically active substances are known including dietary components (alcohol, caffeine), herbal remedies, and newer drugs, i.e., neprilysin inhibitors or SGLT2 inhibitors.

We propose to replace the traditional drug group term diuretics by designations reflecting their molecular targets. So far, this is only applicable for carbonic anhydrase inhibitors. Such a mechanism-based nomenclature has already been implemented for various drug classes in the journal *Naunyn–Schmiedeberg’s Archives of Pharmacology* (Seifert and Schirmer 2021b). This nomenclature as presented by Seifert et al. is in accordance with *IUPHAR* nomenclature classification guidelines for biological targets (https://www.guidetopharmacology.org/nomenclature.jsp, accessed 10 December 2021).

Loop diuretics and thiazide diuretics are already included in this proposal for a revised nomenclature (Seifert and Schirmer 2021b). According to the nomenclature, a more precise term for loop diuretics is Na^+^/K^+^/2Cl^−^ cotransporter inhibitors (NKCC inhibitors) and for thiazide diuretics Na^+^/Cl^−^ cotransporter inhibitors (NCC inhibitors). Potassium-sparing diuretics are not yet listed. Depending on target, our suggestion is to replace the term analogously by epithelial Na^+^ channel blocker (ENaCB) and mineralocorticoid receptor antagonist (MCR antagonist). Osmotic diuretics do not have a defined drug target. As they act in all indications via osmosis, osmotic agent would be a simple, but scientifically correct designation.

Besides nomenclature, validity of the traditional group of diuretics is no longer given with respect to currentness and completeness. As precisely analyzed, included drug classes (especially CAIs) can no longer be clearly categorized as diuretics. Drug repurposing is one of the factors that render a clear classification of CAIs as diuretics obsolete. Our analysis illustrates that many more substances are known to be diuretically active. Drugs launched in the last few years have not been included in the diuretic group. Since older drugs do no longer clearly belong to the diuretic group and newer drugs have not been considered, we propose to dissolve this pharmacological group.

## Supplementary Information

Below is the link to the electronic supplementary material.

Supplementary file1 (XLSX 27 KB)

Supplementary file2 (XLSX 15 KB)

Supplementary file3 (XLSX 14 KB)

## Data Availability

The raw data of the literature search (PubMed and clinicaltrials.gov) is amended as supplementary material.
